# Influence of different types of downscaling on a cortical microcircuit model

**DOI:** 10.1186/1471-2202-14-S1-P112

**Published:** 2013-07-08

**Authors:** Sacha J van Albada, Sven Schrader, Moritz Helias, Markus Diesmann

**Affiliations:** 1 Institute of Neuroscience and Medicine (INM-6) and Institute for Advanced Simulation (IAS-6), Jülich Research Centre and JARA, Jülich, D-52425, Germany; 2 Kirchhoff Institute for Physics, University of Heidelberg, D-69120, Germany; 3 Faculty of Medicine, RWTH Aachen University, D-52062, Germany

## 

Neural network models are routinely downscaled in terms of numbers of neurons or synapses because of a lack of computational resources or the limited capacity of a given neuromorphic hardware. Unfortunately the downscaling is often performed without explicit mention of the limitations this entails [1]. This is relevant since downscaling can substantially affect the dynamics. For instance, reducing the number of neurons N while preserving in-degrees K increases shared inputs and hence correlations.

Theoretical results on scaling are derived using simplifying assumptions. Therefore we use simulations to systematically investigate the effects of downscaling on the dynamics of a layered microcircuit model of early sensory cortex [2]. The model consists of eight excitatory and inhibitory populations of leaky integrate-and-fire point neurons with current-based synapses and homogeneous Poisson input. The full-scale network, comprising approximately 80,000 neurons and 0.3 billion synapses, is in the balanced state [3,4], displaying asynchronous irregular activity. For networks of binary or integrate-and-fire neurons in this state, the total current-based synaptic input to a given neuron is well approximated by a Gaussian noise, of which the mean and variance determine the firing rate. One way of preserving this mean and variance has been touched upon: reducing N but keeping K constant. For reduced K, firing rates can be preserved by an appropriate choice of synaptic weights, the ratio between excitatory and inhibitory weights, and mean external input [3,4].

However, these scaling methods are based on single-neuron dynamics and ignore network effects. For instance, scaling synaptic weights J and mean external input to preserve rates implies constant J^2 ^K [3], which changes the feedback on the population level and hence qualitatively changes the network state [5]. An alternative that takes into account both single-neuron and network effects is to choose J so that J K is constant and adjusting the external drive to maintain the input variance. This preserves the shape of cross-correlation functions in balanced E-I networks [5]. However, the magnitude of the correlations increases as K decreases, and a restriction to positive external input variance limits the compensation for increased intrinsic variance.

We consider the effects of the above scaling options on correlations, firing rates, synchrony, and irregularity of spiking activity. Although each method achieves near-constancy of one or more dynamical features, none preserves all of them. These results can be used to downscale networks of neurons with current-based synapses in the asynchronous irregular regime in a controlled manner. However, since it is not yet possible to analytically predict all the effects of scaling, it remains essential to compare down-scaled simulations with their full-scale counterparts.
